# Amelioration of Compound 48/80-Mediated Itch and LL-37-Induced Inflammation by a Single-Stranded Oligonucleotide

**DOI:** 10.3389/fimmu.2020.559589

**Published:** 2020-09-30

**Authors:** Aleksandra Dondalska, Elin Rönnberg, Haisha Ma, Sandra Axberg Pålsson, Elin Magnusdottir, Tianle Gao, Lucille Adam, Ethan A. Lerner, Gunnar Nilsson, Malin Lagerström, Anna-Lena Spetz

**Affiliations:** ^1^Department of Molecular Biosciences, The Wenner-Gren Institute, Stockholm University, Stockholm, Sweden; ^2^Immunology and Allergy Division, Department of Medicine, Karolinska Institutet, Karolinska University Hospital, Solna, Sweden; ^3^Department of Neuroscience, Uppsala University, Uppsala, Sweden; ^4^Department of Dermatology, Cutaneous Biology Research Center, Massachusetts General Hospital/Harvard Medical School, Boston, MA, United States; ^5^Department of Medical Sciences, Uppsala University, Uppsala, Sweden

**Keywords:** itch, inflammation, mast cells, rosacea, single-stranded oligonucleotides, compound 48/80, MRGPRX2, LL-37

## Abstract

Numerous inflammatory skin disorders display a high prevalence of itch. The Mas-related G protein coupled receptor X2 (MRGPRX2) has been shown to modulate itch by inducing non-IgE-mediated mast cell degranulation and the release of endogenous inducers of pruritus. Various substances collectively known as basic secretagogues, which include inflammatory peptides and certain drugs, can trigger MRGPRX2 and thereby induce pseudo-allergic reactions characterized by histamine and protease release as well as inflammation. Here, we investigated the capacity of an immunomodulatory single-stranded oligonucleotide (ssON) to modulate IgE-independent mast cell degranulation and, more specifically, its ability to inhibit the basic secretagogues compound 48/80 (C48/80)-and LL-37 *in vitro* and *in vivo*. We examined the effect of ssON on MRGPRX2 activation *in vitro* by measuring degranulation in a human mast cell line (LAD2) and calcium influx in MRGPRX2-transfected HEK293 cells. To determine the effect of ssON on itch, we performed behavioral studies in established mouse models and collected skin biopsies for histological analysis. Additionally, with the use of a rosacea mouse model and RT-qPCR, we investigated the effect on ssON on LL-37-induced inflammation. We reveal that both mast cell degranulation and calcium influx in MRGPRX2 transfected HEK293 cells, induced by the antimicrobial peptide LL-37 and the basic secretagogue C48/80, are effectively inhibited by ssON in a dose-dependent manner. Further, ssON demonstrates a capability to inhibit LL-37 and C48/80 activation *in vivo* in two mouse models. We show that intradermal injection of ssON in mice is able to block itch induced via C48/80 in a dose-dependent manner. Histological staining revealed that ssON inhibits acute mast cell degranulation in murine skin treated with C48/80. Lastly, we show that ssON treatment ameliorates LL-37-induced inflammation in a rosacea mouse model. Since there is a need for new therapeutics targeting non-IgE-mediated activation of mast cells, ssON could be used as a prospective drug candidate to resolve itch and inflammation in certain dermatoses.

## Introduction

Numerous inflammatory skin disorders exhibit an increased or dysregulated expression of antimicrobial peptides, which is often associated with pro-inflammatory, innate immune responses (1). For instance, the antimicrobial peptide LL-37, the only member of the human cathelicidin family, has been implicated in the pathogenesis of both atopic dermatitis (2) and psoriasis (3). Additionally, an elevated presence of LL-37 has not only been reported in the lesional skin of rosacea patients but has also been linked to the disease pathology and induction of inflammation in the skin of mice (4). Correspondingly, itch, or pruritus, also presents as a dominant symptom in a multitude of cutaneous disorders (5, 6), and elicits the desire or reflex to scratch, which often exacerbates the disease (7). The differential induction of itch is due to a vast and diverse assortment of pruritogens, which are substances that induce itch-mediated signaling by interacting with cellular detectors [i.e., G protein-coupled receptors (GPCR), ion channels] expressed mainly on nerve fibers (6). At least two distinct primary afferent nociceptive pathways can transduce itch. The histamine-dependent pathway, or histaminergic itch, relies on nerve fibers that can respond to histamine, while the histamine-independent pathway, or non-histaminergic itch, involves nerve fibers that cannot respond to histamine and can also be defined as such based on its lack of response to anti-histamine medications (8, 9). Histamine, which is one of the most well-characterized endogenous pruritogens (10), is predominantly stored in mast cell (MC) secretory vesicles.

MCs are tissue-resident, long-lived innate immune cells, which are predominately and strategically located at barriers exposed to the exterior environment, such as the skin and the respiratory tract. MCs are densely packed with secretory granules containing various pre-formed mediators, including MC specific proteases, which they can rapidly release in response to allergens, infectious agents, and toxins. Further, MC degranulation is often accompanied by the *de novo* generation of various products including prostaglandins (PG)s, chemokines, and cytokines (11–13). Classically, MCs are activated by the cross-linking of high-affinity Immunoglobulin E (IgE) receptors (FcεRI), however, they can also be stimulated independently of IgE (11, 14). Notably, MCs have been shown to express the GPCR Mas-related G protein-coupled receptor X2 (MRGPRX2), which is highly and selectively expressed in human skin MCs (15). MRGPRX2 (and the mouse ortholog Mrgprb2) has been revealed to be the receptor to a wide range of cationic substances, collectively known as basic secretagogues, which comprise antimicrobial peptides (i.e., LL-37), neuropeptides, certain drugs (i.e., opioids, antibiotics), and the widely used MC activator C48/80 (16). These basic secretagogues can trigger MRGPRX2 and thereby induce pseudo-allergic reactions characterized by histamine and protease release, inflammation, and pruritus (17). Furthermore, MRGPRX2, including downstream signaling, may be involved in a plethora of skin diseases. For instance, MRGPRX2 has been shown to be expressed at increased levels in skin MCs of patients with severe chronic urticaria, or hives (18). Additionally, Meixiong et al. have recently demonstrated that MrgprB2, the mouse ortholog of MRGPRX2, was critical for itch in allergic contact dermatitis (ACD), concluding that MRGPRX2 may be a target for ACD-associated itch in humans (19).

Oligonucleotides were previously shown to have immunosuppressive properties in the skin. Grimstad et al. demonstrated that oligonucleotides could inhibit the pro-inflammatory response induced by the activation of Toll-like receptor 3 (TLR3) in primary keratinocytes (20), while Dorn et al. observed that a 16 nucleotide-long oligonucleotide could suppress the secretion of the pro-inflammatory cytokine interleukin-8 (IL-8) in a human keratinocyte cell line (21). We recently found that certain single-stranded oligonucleotides have the ability to inhibit endocytic pathways used by cargo destined for TLR 3/4/7 signaling endosomes in human monocyte-derived dendritic cells (MoDCs) (22). Both ssDNA and ssRNA conferred the endocytic inhibition, it was concentration dependent, and required a certain oligonucleotide length but was not strictly dependent on sequence. We thus decided to investigate the possibility that single-stranded oligonucleotides could modulate MGRPRX2-mediated MC degranulation and itch, a downstream effect of such degranulation, and inflammation using an established immunomodulatory 35 nucleotide-long single-stranded oligonucleotide (denoted as ssON) (22, 23).

We present *in vitro* data revealing that ssON blocks the activation of MRGPRX2 by the basic secretagogue C48/80 and the antimicrobial peptide LL-37 in human mast cells. Additionally, we provide evidence that ssON suppresses itch induced by C48/80 in mice by inhibiting the acute degranulation of skin MCs, as well as, LL-37-mediated inflammation in a murine model of rosacea. By demonstrating the inhibitory effect of ssON on non-IgE-mediated itch and MC degranulation, we report a novel immunomodulatory potential of oligonucleotides in the context of the pathophysiology of itch and perhaps certain inflammatory skin disorders.

## Results

### ssON Blocks Mast Cell Degranulation Induced by C48/80 and LL-37 *in vitro*

**Figure 1:**
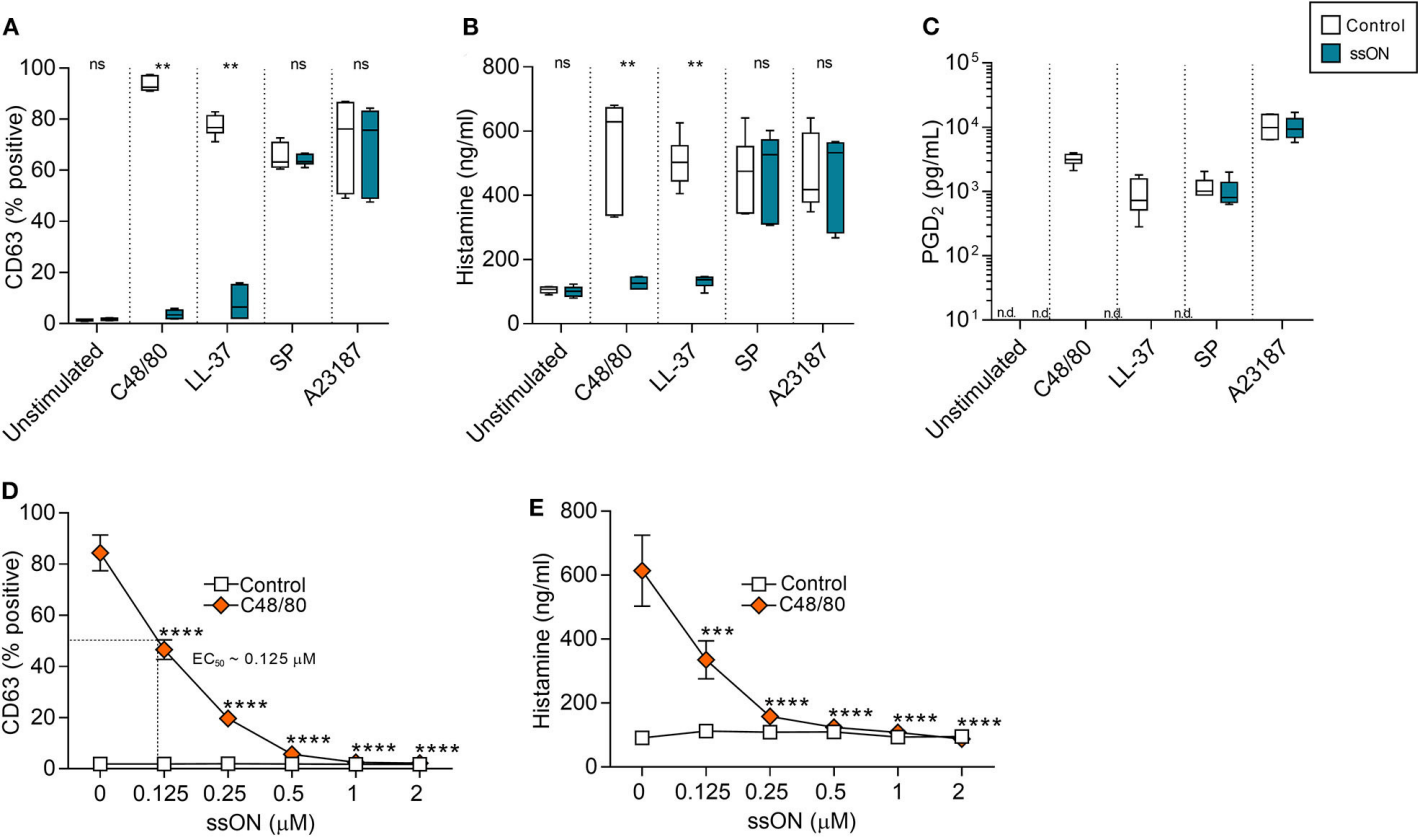
ssON blocked mast cell degranulation induced by C48/80 and LL-37. Degranulation of LAD2 cells was measured by the upregulation of CD63 on the cell surface and histamine and PGD2 release. **(A–C)** LAD2 cells were stimulated with C48/80 (1 μM), LL-37 (10 μg/mL), SP (0.5 μM), or A23187 (2 μM) with and without ssON (0.5 μM) for 30 min. ssON significantly blocked C48/80- and LL-37-induced degranulation as shown by the reduced **(A)** CD63 frequency, **(B)** histamine release, and **(C)** PGD2 release, but had no effect on cells stimulated with SP or A23187. **(D,E)** The ssON-mediated inhibition of C48/80-induced degranulation in LAD2 cells was concentration-dependent (EC50 around 0.125 μM). Statistical significance was assessed by non-parametric Mann-Whitney test for **(A,B)** and two-way ANOVA test for **(D,E)**. *P*-value: ****P < 0.0001, ***P = 0.0001, **P = 0.0022, ns, non-significant. Data is shown as mean ± SEM. n.d., not detectable.

MC degranulation can be induced by either IgE-dependent or –independent pathways, therefore, both types of activation were examined. To determine the effect of ssON on IgE-independent degranulation, we treated a human MC line, LAD2, with a variety of degranulating compounds, with or without ssON (Figure 1). These compounds included: C48/80, a potent, synthetic MC degranulator extensively used in field; LL-37, a naturally occurring antimicrobial peptide of high interest due its potential involvement in a variety of skin disease including rosacea and psoriasis; substance P (SP), a neuropeptide associated with itch and inflammation; and A23187, a calcium ionophore. We assessed MC degranulation by evaluating the expression of CD63 (Supplementary Figure 1), a surface molecule which has been identified as an activation marker for mast cells that is also upregulated very rapidly after allergen challenge (24). Additionally, we evaluated the secretion of both histamine and prostaglandin D_2_ (PGD_2_), a pro-inflammatory lipid mediator primarily released from mast cells (25). Both C48/80- and LL-37-induced degranulation was significantly inhibited by ssON treatment as observed by the substantial reduction of CD63 expressing cells (Figure 1A), histamine release (Figure 1B), and PGD_2_ release (Figure 1C). This effect was dose-dependent with an EC_50_ of ~0.125 μM (Figures 1D,E). However, ssON had no impact on degranulation induced by either SP or the calcium ionophore A23187 (Figures 1A–C). In order to determine if the inhibitory activity observed was a feature for the immunomodulatory 35 nucleotide(nt) long oligonucleotide ssON, we tested whether ON15, a 15 nt long single-stranded oligonucleotide previously shown not to have an immunosuppressive effect in MoDCs (22), could also display a similar result on MC degranulation. Accordingly, LAD2 cells were treated with C48/80, with or without ssON or ON15, and assessed for degranulation. Only the active 35 nt oligonucleotide, ssON, significantly blocked C48/80-induced degranulation, as shown by the reduced CD63 frequency, while the inactive oligonucleotide ON15 had no significant effect on the degranulation (Supplementary Figure 2), substantiating that the inhibitory effect on non-IgE-mediated degranulation observed occurs for 35 nt ssON and not a general ability of oligonucleotides. In regard to IgE-mediated degranulation, we did not detect any inhibitory activity by ssON in cord blood-derived mast cells (CBMCs) or LAD2 (Supplementary Figure 3). These data demonstrate that ssON can inhibit MC degranulation induced by C48/80 and LL-37 in LAD2 cells, suggesting an effect on MRGPRX2-mediated degranulation.

### ssON Inhibits the Activation of MRGPRX2 Induced by the Agonists LL-37 and C48/80

**Figure 2:**
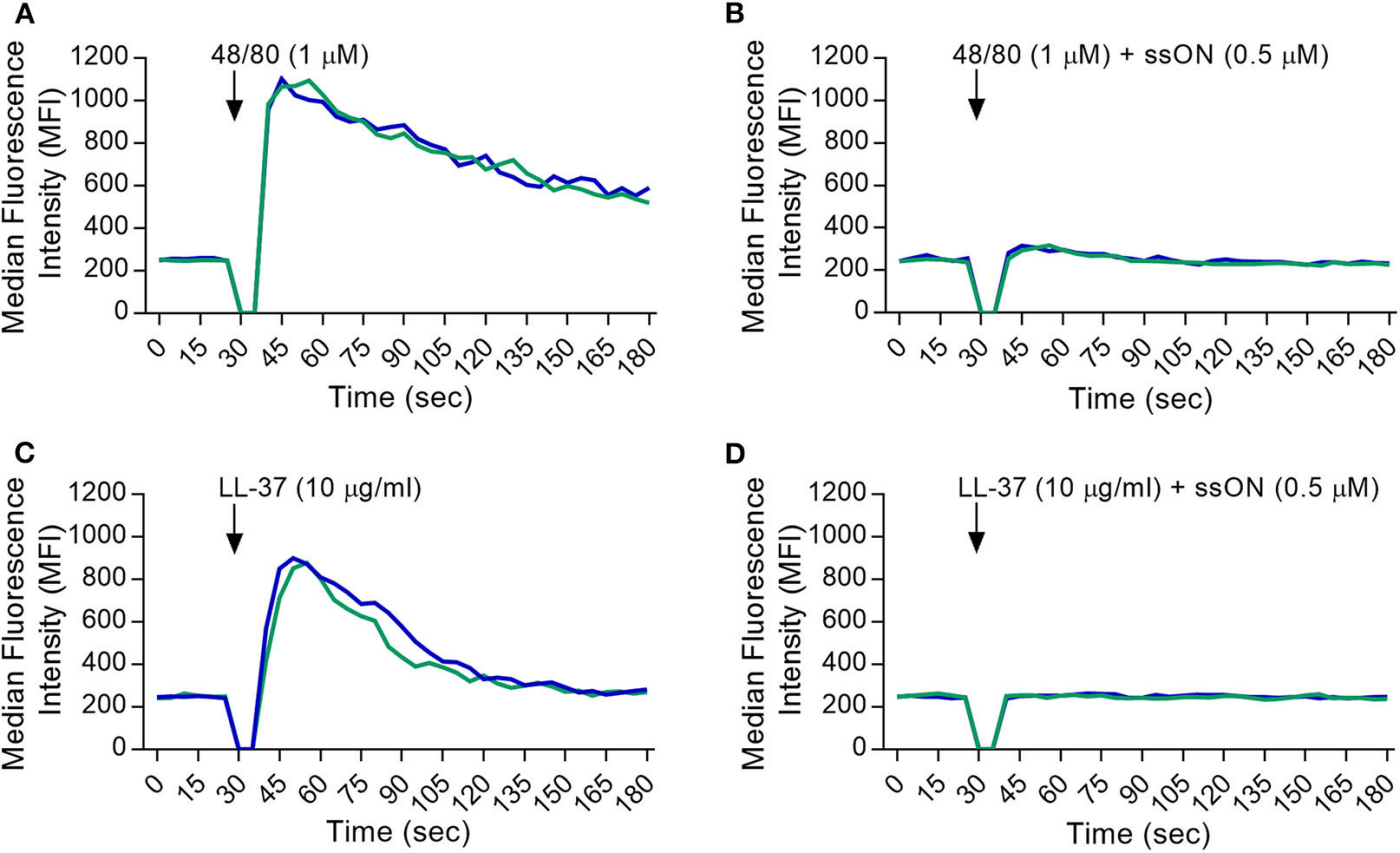
ssON inhibited the activation of MRGPRX2 by C48/80 and LL-37. A stably MRGPRX2-transfected HEK293 cell line was used. Intracellular calcium (Ca^2+^) influx was determined by loading cells with Fluo-3 dye and measuring the median fluorescence intensity (MFI) during treatment. MFI curves were recorded for 30 s (sec) to obtain a baseline. The cells were then stimulated during the gap in the analysis with **(A,B)** C48/80 or **(C,D)** LL-37 with and without ssON, and further recorded for 2.5 min. The Ca^2+^ influx induced by the activation of MRGPRX2 with C48/80 and LL-37 was completely blocked by ssON. Each curve represents an independent sample. Gaussian smoothing is applied. Three independent experiments were performed in duplicate. A representative result is shown.

To provide more insight into the selectivity of ssON's receptor interactions, we utilized a stably MRGPRX2-transfected HEK293 cell line to further assess the inhibitory effect of ssON. The activation of the MRGPRX2 receptor was evaluated by loading cells with a fluorescently labeled calcium indicator, Fluo-3, and using flow cytometry analyses to measure the influx of intracellular calcium (Ca^2+^). A non-MRGPRX2 expressing HEK293 cell line (HEK293 null) was used as a negative control. Ca^2+^ influx was measured in cells treated with the basic secretagogues LL-37 and C48/80, with and without the presence of ssON. C48/80 and LL-37 both induced a strong Ca^2+^ influx in the MRGPRX-2 transfected HEK293 cells (Figures 2A,C, respectively) as observed by the peak in the median fluorescence intensity (MFI). We found that the addition of ssON treatment was able to abolish the signal produced by C48/80- (Figure 2B) and LL-37- (Figure 2D) induced Ca^2+^ influx. Moreover, the observed block of LL-37-induced Ca^2+^ influx by ssON was concentration-dependent (Supplementary Figure 4). The HEK293 null control cells did not mount any influx of Ca^2+^ after treatment with C48/80 treatment but displayed a moderate Ca^2+^ influx after LL-37 treatment, which was also inhibited by ssON (Supplementary Figure 5). The Ca^2+^ influx observed in the LL-37-treated HEK293 null cells was not completely unexpected as LL-37 has been associated with a plethora of effects on several different receptors (26). Interestingly, ssON appeared to have no effect on Ca^2+^ influx induced in MRGPRX2-transfected cells treated with either morphine or ZINC-3573, a selective agonist probe for MRGPRX2 (Supplementary Figure 6). Additionally, ssON had no effect on Ca^2+^ signaling induced in either HEK293 cell line when cells were treated with ionomycin (1 μg/ml), a potent and widely used inducer of calcium influx (data not shown). Altogether, these findings reveal that ssON has the capacity to inhibit MRGPRX2 activation induced by C48/80 and LL-37.

### ssON Can Ameliorate Histamine-Related Itch Induced by C48/80 *in vivo*

**Figure 3:**
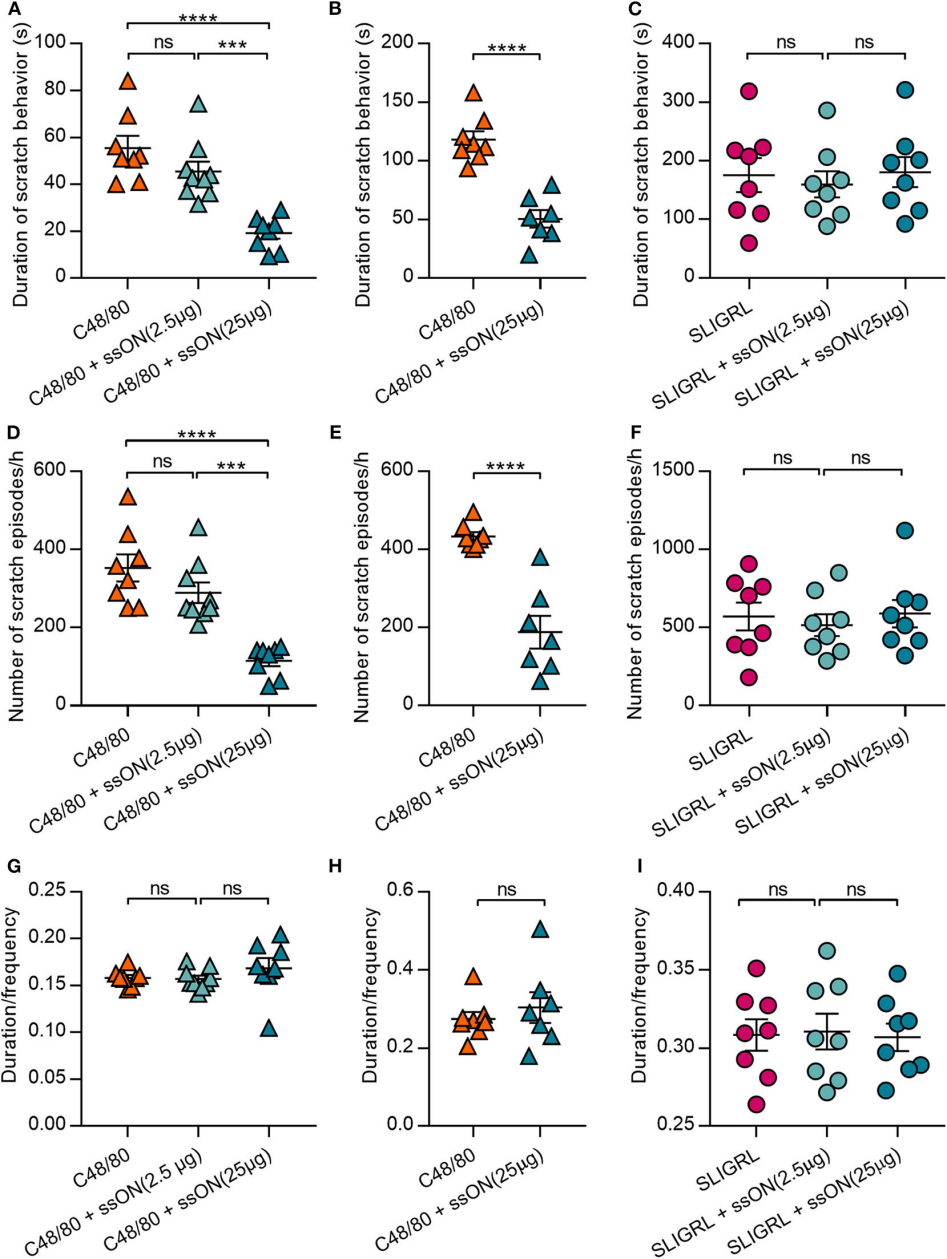
C48/80-induced scratch behavior was inhibited by ssON in a concentration dependent manner, while PAR2-mediated scratch behavior was unaffected. **(A–C)** Duration of scratch behavior (seconds), **(D–F)** frequency of scratch behaviors, and **(G–I)** the mean length per scratch behavior (duration/frequency) were recorded for 1 h immediately following treatment in two separately performed experiments (**A,C,D,F,G,I** and **B,E,H**, respectively). Histamine-related scratch behavior was induced by intradermal injection of 50 μL of either C48/80 (10 μg) alone or a combination of C48/80 (10 μg) and ssON (25 μg or 2.5 μg). Non-histamine-related scratch behavior was induced by intradermal injection of 50 μL of either SLIGRL (100 μg) alone or a combination of SLIGRL (100 μg) and ssON (2.5 or 25 μg). Statistical significance was assessed by one-way ANOVA or unpaired, two-tailed *t*-test for **(B, E,H)**. All data passed Shapiro-Wilk's normality test (GraphPad Prism); *N* = 7–9 mice. *P*-value: ****P < 0.0001, ***P = 0.0002–0.0005, ns, non-significant. Data is shown as mean ± SEM.

Basic secretagogues have been shown to activate mouse mast cells through Mrgprb2, the ortholog of MRGPRX2(16). Hence, we sought to determine if we could translate the observed *in vitro* inhibitory effect of ssON to an *in vivo* setting. We firstly investigated whether ssON can affect itch induced by C48/80, which has been widely used to study histamine-related itch (27). Further, we assessed the effect of ssON treatment on both histamine-related and -unrelated scratch behavior in wild type BALB/c mice. The effect on non-histaminergic itch was evaluated using the protease-activated receptor 2 (PAR2) agonist SLIGRL, an itch-inducing agent widely used to study histamine-unrelated itch as it cannot be blocked by histamine H1 receptor antagonists (28–30). Mice were injected intradermally with the pruritogens C48/80 or SLIGRL, with or without ssON of two different concentrations (2.5 or 25 μg), into the nape of the neck. The scratch duration (total length of scratch episodes) and frequency (number of scratch episodes) were recorded for 1 h immediately following the injection (Figure 3). Notably, the duration and scratch frequency induced by C48/80 were both reduced by ssON in a concentration dependent manner in two separately performed experiments (Figures 3A,B,D,E, respectively), where the higher concentration of ssON significantly inhibited C48/80-induced scratch behavior. The ssON had no effect on scratch duration and scratch episodes induced by SLIGRL (Figures 3C,F) as the scratch behavior remained unaffected when ssON treatment was added. Scratch intervals (duration/frequency) were not affected by either high or low dose ssON treatment (Figures 3G–I). These data provide evidence that ssON can ameliorate acute C48/80-dependent itch, as shown by the inhibition of C48/80-mediated pruritus but is unable to affect histamine-unrelated itch induced by the PAR2 agonist SLIGRL.

### Acute Skin Mast Cell Degranulation Is Inhibited by ssON

**Figure 4:**
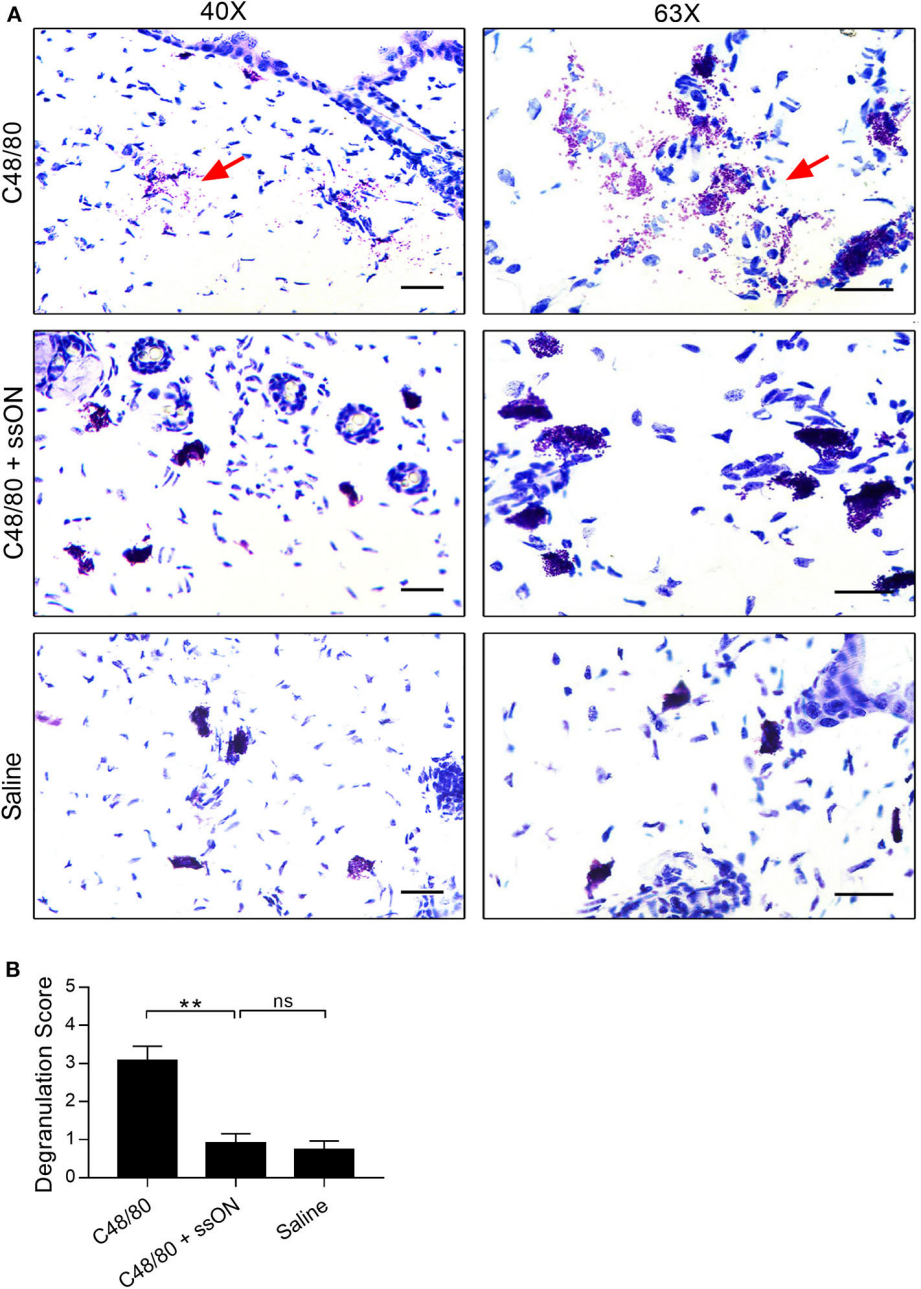
Decreased C48/80-induced mast cell degranulation in mouse skin with ssON treatment. Skin biopsies were taken from mice 1 h post-treatment at the injection site, and then stained with Toluidine Blue. Each sample was graded for mast cell degranulation and given a score using a scale from 0 to 4: a score of 0 was given for intact mast cells; 1, for mostly intact; 2, for mild degranulation; 3, for moderate degranulation; and 4, for heavy degranulation. **(A)** Representative histological sections and **(B)** the average scores are shown for each treatment group. An average score of 3.0 was determined for C48/80 (10 μg) treatment, a score of 0.9 was determined for the combination treatment of C48/80 and ssON (10 and 25 μg, respectively), and a score of 0.8 was determined for saline treatment. Left panels, magnification at 40X; right panels, magnification 63X. All scale bars represent 10 μm. Red arrows point to degranulation events. N=6 for each group. Statistical significance was assessed by non-parametric Mann-Whitney test. *P*-value: **P < 0.01, ns, non-significant. Data is shown as mean ± SEM.

We next investigated MC related events occurring *in vivo* after ssON treatment, to get insights as to how ssON may modulate C48/80-mediated acute itch in mice. C48/80 induces itch by initiating rapid MC degranulation and thus causing the release of pre-formed vesicles containing histamine and proteases, which in turn activate afferent neurons (27). Therefore, we assessed the effect of ssON on the degree of MC degranulation in murine skin treated with C48/80 *in vivo*. Skin biopsies were taken at the injection site of mice treated intradermally with C48/80 (10 μg), with or without ssON (25 μg), or with saline. The biopsies were subsequently sectioned, stained with Toluidine Blue, evaluated, and given a score from 0 to 4 for the level of MC degranulation present (Figure 4). The addition of ssON to C48/80 treatment significantly reduced the level of acute MC degranulation in murine skin (Figure 4B). Tissue injected with a mixture of ssON and C48/80 showed a clear decrease in MC degranulation, while extensive MC degranulation was observed in tissue injected with C48/80 alone (Figures 4A,B). Mild to no degranulation was observed in tissue treated with saline (Figure 4). Moreover, the degree of degranulation in skin injected with saline was comparable to that observed with the combination treatment of ssON and C48/80. Altogether, these data demonstrate that ssON can ameliorate C48/80-mediated itch in mice by inhibiting acute MC degranulation induced by C48/80 in the skin.

### ssON Ameliorates LL-37-Mediated Inflammation in a Murine Model of Rosacea

**Figure 5:**
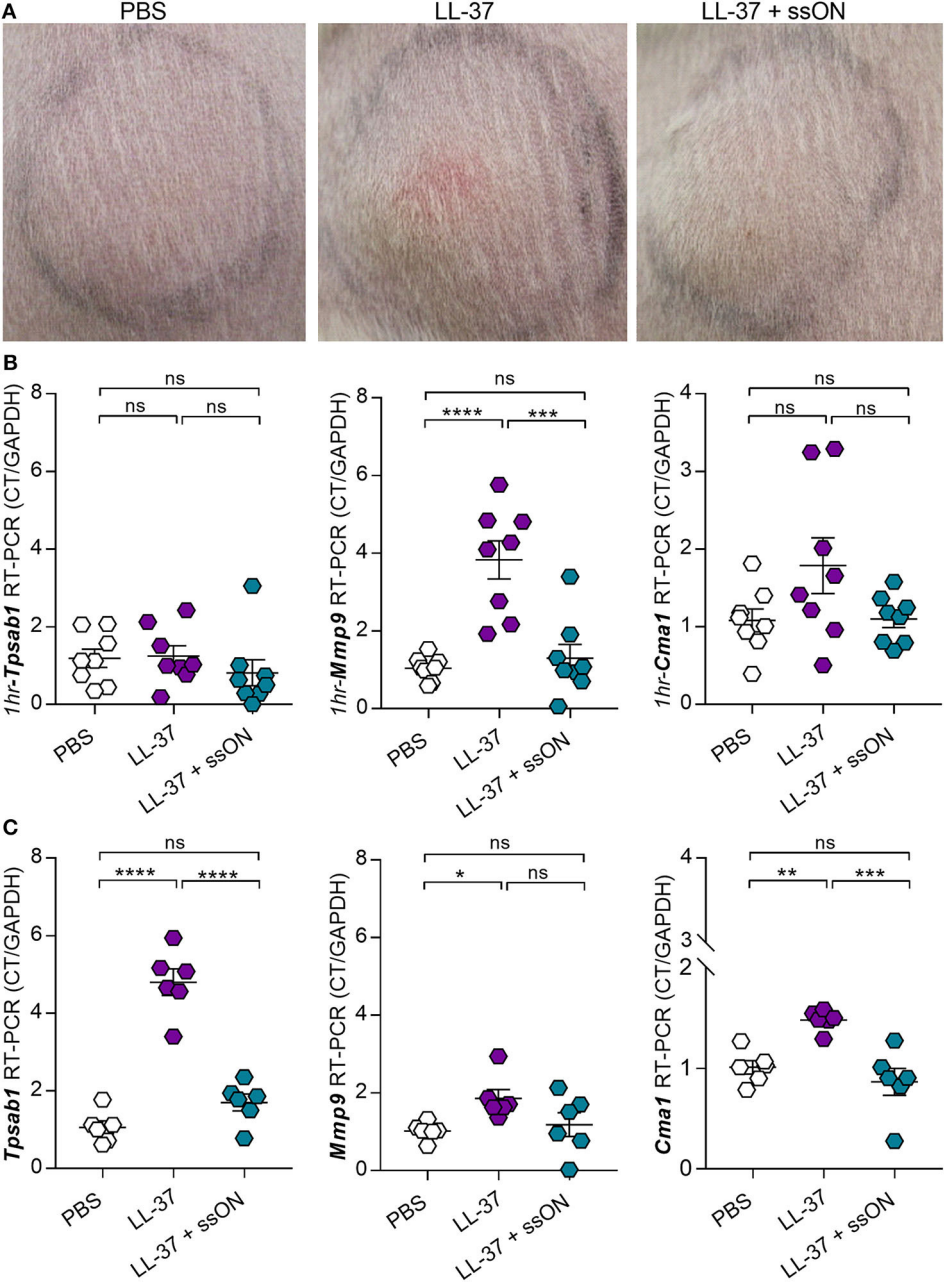
ssON ameliorated LL-37-mediated inflammation in a rosacea mouse model. Mice were treated subcutaneously in the skin of the back with LL-37 (320 μM), with or without ssON (57 μg), or PBS. **(A)** Photographs for the respective treatments were taken at the clinical endpoint, 72 h after the induction of rosacea-like inflammation. The expression of mouse chymase (Cma1), tryptase (Tpsab1), and matrix metalloproteinase-9 (Mmp9) in the skin was assessed using RT-PCR in biopsies collected **(B)** 1 h post treatment or **(C)** 72 h after observation. Representative pictures are shown for each treatment group. Statistical significance was assessed by one-way ANOVA or Kruskal-Wallis test for Tpsab1 expression in C and Mmp9 expression in B. Normality was assessed using the Shapiro-Wilk normality test (GraphPad Prism); *N* = 8 for **(B)** and *N* = 6 for **(C)**. *P*-value: ****P < 0.0001, ***P = 0.0002–0.0006, **P = 0.0057, *P = 0.033, ns, non-significant. Data is shown as mean ± SEM. 1 h = 1 hour post treatment.

Given that we observed an inhibitory effect with ssON in the evaluation of C48/80-meditead itch, we subsequently investigated if ssON could similarly modulate LL-37 *in vivo*. Since LL-37 does not exhibit any pruritogenic capacity (31), we used a well-established rosacea mouse model (4, 32) to investigate whether ssON could inhibit LL-37-mediated inflammation *in vivo*. As previously described, wild type Balb/c mice were injected subcutaneously with LL-37 (320 μM), with or without ssON (57 μg), or PBS twice a day for 2 days. Photographs were taken at the clinical endpoint, which was 72 h from the initial treatment, and revealed that we were indeed able to induce LL-37-mediated inflammation, which appeared to be reduced with ssON treatment, as seen by the diminished redness at the injection site (Figure 5A). Mast cells have previously been shown to be key mediators in LL-37-induced inflammation in rosacea (32). Hence, we evaluated the expression levels of the MC specific proteases chymase and tryptase in the skin at the injection site using the genes *Chymase 1* (*Cma1*) and *Tryptase alpha/beta 1* (*Tpsab1*), as well as, the levels of *Metalloproteinase 9* (*Mmp9*), which has been shown to be secreted by mast cells and keratinocytes after histamine stimulation (33, 34). Therefore, to determine if ssON could modulate the LL-37-mediated activation of skin mast cells, we analyzed the effect of ssON treatment on the mRNA levels of *Cma1, Tpsab1*, and *Mmp9* in skin samples taken either immediately 1 h after one treatment (Figure 5B) or at the clinical endpoint (Figure 5C). No differences in the mRNA levels of *Tpsab1* or *Cma1* were detected at the earlier time point of 1 h between the different groups; however, *Mmp9* expression was significantly higher in the LL-37 treated group compared to PBS and completely inhibited when treated with ssON (Figure 5B). Conversely, all three genes were upregulated at the clinical endpoint, with *Mmp9* to a lesser extent, and were downregulated by ssON treatment (Figure 5C). These data provide evidence that ssON treatment is able to dampen the upregulation of *Tpsab1, Mmp9*, and *Cma1* in murine skin induced by LL-37 and subsequently reduces the LL-37-mediated inflammation in a murine model of rosacea.

## Discussion

A 35 nt ssON was previously shown to modulate cytokine production from cells located in skin such as keratinocytes *in vitro* (23) and upon intradermal injection *in vivo* in macaques (22). We further found that ssON has the capacity to inhibit certain endocytic uptake routes utilized by ligands destined for TLR3/4 and 7 expressing endosomes, thereby preventing TLR signaling in human monocyte-derived dendritic cells and peripheral blood mononuclear cells (22). However, to our knowledge, the functional capacity of ssON, or other oligonucleotides, to modulate MC activation in the skin has not been assessed previously. Herein, we have demonstrated that ssON has the ability to selectively suppress certain non-IgE mediated MC degranulation, thereby ameliorating downstream events including itch and inflammation.

Although human MCs display considerable heterogeneity, both phenotypically and functionally (35), they are divided into two general subtypes based on the protease content of their granules (36). Connective tissue MCs express tryptase, chymase, carboxypeptidase, and cathepsin G and are classified as tryptase- and chymase-expressing MCs (MC_TC_) (37), while the majority of gut and lung MCs only express tryptase and are hence classified as tryptase-expressing mast cells (MC_T_) (36, 38). MRGPRX2 is highly expressed in human skin MC_TC_, but not in lung MC_T_ (18, 39) and thus, only MC_TC_ are able to respond to basic secretagogues. Since mast cells are tightly integrated into the tissue they reside in, making their isolation quite challenging (40), we utilized LAD2 cells, the only human MC line expressing MRGRPRX2 (18), to investigate the effect of ssON on MRGPRX2-mediated MC degranulation. By analyzing the expression of the degranulation marker CD63 and the secretion of both PGD_2_ and histamine in LAD2 cells, we observed that ssON significantly and efficiently (EC_50_ ~ 125nM) inhibited MC degranulation induced not only by C48/80, but also the antimicrobial peptide LL-37. Furthermore, no inhibitory effect by ssON was observed with the calcium ionophore A23187, which can penetrate the cell membrane resulting in Ca^2+^ influx and degranulation (41). These data show that ssON does not prevent degranulation *per se*. We hypothesized that it would be unlikely for ssON to affect IgE-mediated degranulation as it involves the aggregation of high affinity FcεRI receptors through crosslinking of IgE bound to antigen (11). Nonetheless, we assessed the effect of ssON on IgE-mediated degranulation in human MCs. As expected, ssON had no effect on IgE-mediated degranulation in CBMCs or LAD2.

Since we observed such a potent inhibition using ssON *in vitro*, we sought to determine if we could observe a similar capacity of ssON *in vivo* in mice. Basic secretagogues have been shown to activate human MC_TC_ and mouse connective tissue MCs via MRGPRX2 and Mrgbr2, respectively, thereby inducing pseudoallergic reactions in both humans and mice (16). Hence, we evaluated established mouse models that involved the use of C48/80, known to induce itch, and LL-37, known to induce inflammation. We first investigated the effect of ssON on acute, non-IgE-mediated pruritus in mice (BALB/c) by injecting pruritogens intradermally into the nape of the neck, as previously described (42, 43), and analyzing both histamine-related and -unrelated itch. We observed that ssON treatment had a significant inhibitory effect on histamine-related scratch behavior induced by C48/80, similar in antagonizing capacity to that observed by Azimi et al. using the tripeptide QWF (44), where they instead used the mouse cheek model of acute itch. C48/80 activates on MCs and induces the release of vesicular contents including histamine and proteases, thereby causing itch in both mice and humans (16). However, C48/80 has also been shown to directly interact with sensory nerves, including primary dorsal ganglion (DRG) cells (45). To gain further insight into the mechanisms involved, we performed histopathological evaluation of skin biopsies collected at the injection site. Toluidine blue staining revealed extensive degranulation after injection with C48/80 as expected (46), while the level of MC degranulation observed with the combination treatment of C48/80 and ssON was comparable to that observed with saline treatment. It remains to be elucidated whether ssON also has a direct effect on sensory nerves, such as DRG and enteric neurons, but skin histological staining data confirm that ssON can inhibit C48/80-induced itch by blocking C48/80-mediated MC degranulation in mouse skin.

We subsequently investigated ssON's capacity to modulate inflammation induced by LL-37 in a mouse model of rosacea. This *in vivo* model was selected since, unlike C48/80, LL-37 has not been reported to have any pruritogenic capacity nor does not seem to display any itch-inducing activity in mice (47). The latter has been demonstrated by Reddy et al. who showed that LL-37 indeed induced calcium influx in MRGPRX2 and MrgprB2 transfected cells but lacked an ability to provoke scratching behavior in a mouse cheek model of acute itch. It is possible that the lack of itch from LL-37 is due to differential signaling associated with receptor activation. Although our data clearly demonstrate that ssON has the potent capacity to inhibit C48/80-mediated activation of mast cells, the discovery of ssON's capacity to modulate LL-37 is of more biological significance. LL-37 is a multifaceted immunomodulatory compound involved in a plethora of processes, ranging from inflammation, wound healing, and angiogenesis (48), that can be secreted by certain immune cells including mast cells, which are a main producer, and neutrophils, as well as keratinocytes (1). Notably, LL-37 can induce the degranulation of mast cells, which can in turn release histamine, cytokines, and various inflammatory mediators and can thus create an inflammatory milieu in the dermis by additionally activating keratinocytes and supporting the migration of neutrophils to the epidermis (1). Our data revealed that ssON could effectively inhibit the LL-37 mediated inflammation in the skin of mice and reduced the gene expression of the MC specific tryptase and chymase. Due to LL-37's implications in a variety of inflammatory dermatoses including psoriasis, atopic dermatitis, and rosacea, its modulation by ssON could provide an option in a therapeutic setting.

To gain further insight into whether ssON's inhibitory effect is specific for MRGPRX2, we used a MRPGPRX2-transfected HEK293 cell line and measured Ca^+2^ influx upon ligand binding. We observed that ssON was able to completely abolish Ca^+2^ influx induced by both compound 48/80- and LL-37-mediated activation of MRGPRX2 in a dose-dependent manner. However, we observed a complete lack of an inhibitory effect by ssON in MRGPRX2-transfected HEK293 cells after stimulation with two other MRGPRX2 agonists, morphine and ZINC-3573. Given that the purification of GPCRs has been challenging, the current structural information about this type of receptor is limited and crystallography data has often been used in attempts to predict structural properties regarding the regions involved in ligand binding and downstream signaling (37). Lansu et al. performed PRESTO-tango screening for MRGPRX2 ligands and have shown that Glu164 and Asp184 are necessary for opioid activation, however it is unknown if antimicrobial peptides, neuropeptides or other basic secretagogues interact with the same sites (49). Similarly, Reddy et al. found that the peptide QWF inhibits C48/80- induced degranulation but not LL-37 and further showed that a single amino acid E164 in MRGPRX2 is necessary for activation by C48/80 (47). These studies point to the complexity behind the differential agonist-MRGPRX2 binding sites, which also entails the conformational changes predicted to take place upon ligand binding. It is therefore probable that the differential capacity of ssON to inhibit certain MRGPRX2 ligands such as C48/80 and LL-37, but not morphine or ZINC-3573 is due to the presence of different binding sites on MRGPRX2. We can further speculate that ssON cannot affect the opioid-binding pockets as shown by the lack of an inhibitory effect on both morphine and ZINC-357 but could instead affect distinct binding sites utilized by other secretagogues. Consequently, we can conclude that ssON treatment does not prevent the activation of MRGPRX2 by all secretagogues and may act on specific binding sites or downstream signaling events. Future studies are warranted to closely characterize differential ligand-agonist binding properties to facilitate the development of drugs targeting MRGPRX2. Here, we show that ssON can inhibit C48/80- and LL-37-mediated activation of MRGPRX2.

Oligonucleotides were previously shown to have immunosuppressive effects in the context of autoimmunity and inflammation (50–52). Our data have revealed a new immunomodulatory capacity of oligonucleotides in the context of hypersensitivity by demonstrating that ssON, a 35 bases-long non-coding single-stranded DNA, is able to ameliorate IgE-independent itch and inflammation through the modulation of MC degranulation induced by certain basic secretagogues, namely C48/80 and LL-37. SsON has the potential to be utilized in the resolution of itch or inflammation in particular pathological settings in the skin that require interference with non-IgE-mediated activation of MCs.

## Materials and Methods

### Agonists and Oligonucleotides

Synthetic, endotoxin-free, and completely phosphorothioate-modified oligonucleotides, denoted ssON and ON15, were purchased from Integrated DNA Technologies. ssON has the following sequence: 5′-GAAGTTTTGAGGTTTTGAAGTTGTTGGTGGTGGTG-3′. ON15 has the following sequence: 5′-GGTTTTGAAGTTGTT-3′. C48/80, SLIGRL, SP and A23187 were purchased from Sigma-Aldrich. LL-37 was purchased from Bachem and Anaspec.

### Cells and Cell Lines

#### LAD2 Cells

The human LAD2 mast cell line (53) was cultured in StemPro-34 SFM medium (Life Technologies Carlsbad, CA) supplemented with 2 mM L-glutamine, 100 U/ml penicillin, 100 μg/ml streptomycin, and 100 ng/ml recombinant human stem cell factor (Sobi, Stockholm, Sweden). The cells were maintained at a density between 0.5 × 10^6^ and 0.8 × 10^6^ cells/ml at 37°C and 5% CO_2_ and hemi depleted once a week.

#### Cord-Blood-Derived Mast Cells

Cord blood-derived mast cells (CBMCs) were cultured as previously described (53–56). Briefly, mononuclear cells were isolated from fresh cord blood using Ficoll-Paque Plus centrifugation (GE Healthcare). CD34+ cells were then selected from the mononuclear cell fraction using MACS separation columns (LD Columns) and the CD34 human MicroBead kit (Miltenyi Biotech). The CD34+ cells were cultured for 3 weeks in StemPro-34 serum-free medium supplemented with L -glutamine (2 mM), 6 mg/ml penicillin G, 5 mg/ml streptomycin sulfate, recombinant human IL-3 (1 ng/ml; added only the first week; PeproTech), stem cell factor (100 ng/ml; Sobi Stockholm, Sweden), and recombinant human IL-6 (10 ng/ml; PeproTech). Thereafter the cells were cultured in RPMI 1640 medium (Sigma Aldrich) supplemented with 10% fetal calf serum, 0.01 M HEPES, 0.5x non-essential amino acids, 2 mM L-glutamine, 100 units/ml penicillin, 0.1 mg/ml streptomycin and 48 μM β-mercaptoethanol (Sigma Aldrich), stem cell factor (100 ng/ml; Sobi), and recombinant human IL-6 (10 ng/ml; PeproTech). CBMCs were stained for tryptase-like enzymatic activity as a marker of maturity. When the cells were 90% tryptase positive, they were used for experiments (after about 8–10 weeks in culture).

#### HEK293 Cells

The stably MRGPRX2-transfected HEK293 cell line was produced as previously described (44). The cells were cultured at 37°C with 5% CO_2_ in DMEM media (Hyclone) supplemented with 2 mM L-glutamine, 100 units/ml penicillin, 0.1 mg/ml streptomycin (Hyclone), 10% Fetal Bovine Serum (Sigma Aldrich), and 500 μg/ml G418 (Invivogen). Non-transfected HEK293 cells were used as a negative control. The cells were cultured at 37°C with 5% CO_2_ in DMEM media (Hyclone) supplemented with 2 mM L-glutamine, 100 units/ml penicillin, 0.1 mg/ml streptomycin, 0.01 M HEPES (Hyclone), and 10% Fetal Bovine Serum (Sigma Aldrich). Both HEK293 cell lines were maintained at a confluence of ~70% and split accordingly.

### MC Degranulation

#### IgE-Dependent Activation

Degranulation by FcεRI crosslinking was performed in human CBMCs and LAD2 cells. 10 ng/ml of IL-4 (Peprotech) was added 4 days prior and 1 μg/ml of human IgE (Calbiochem) 1 day prior to crosslinking. Cells were placed in PIPES-BSA buffer (PIPES buffer containing 1% bovine serum albumin) at a concentration of 0.2 × 10^6^ cells/ml in a 96 well plate. Cells were cross-linked with 2 μg/ml of anti-IgE antibody (Sigma Aldrich) for CBMCs and 200 μg/ml of anti-IgE antibody for LAD2 cells, with or without ssON (0.5μM). The ionophore A23187 (2 μM) was used as a positive control. Cells were incubated for 30 min at 37°C and 5% CO_2._ The cells were then stained with anti-CD63-PE-Cy7 (BD). Samples were run on a Canto II (BD) and analyzed using the software FlowJo.

#### IgE-Independent Activation

LAD2 cells were placed in PIPES-BSA buffer at a concentration of 0.4 × 10^6^ cells/ml in a 96 well plate and treated with C48/80 (1 or 1.5 μM) or LL-37 (10 μg/ml) with and without ssON (0.5 μM unless shown otherwise). The calcium ionophore A23187 (2 μM) was used as a positive control. Cells were incubated for 30 min at 37°C and 5% CO_2._ Supernatants were collected, and the cells were then stained with Fixable Viability Stain 450 (FVS450; BD) and anti-CD63-PE-Cy7 (or –PE; BD). Samples were run on a Canto II (BD) or Fortessa (BD) and analyzed using the software FlowJo. Histamine released into the supernatant was quantified using a histamine release test according to the manufacturer's instructions (RefLab). Briefly, this test is based on the adsorption of histamine to glass fiber-coated microtiter plates. The glass fibers bind histamine with high affinity and selectivity. The plates were sent to RefLab where the histamine was released and detected fluorometrically (OPA-method) by a HISTAREADER™ 501-1. PGD_2_ released into the supernatant was quantified using a Prostaglandin D_2_-MOX ELISA kit according to the manufacturer's instructions (Cayman Chemical).

### Measurement of Intracellular Calcium

The influx of intracellular calcium was measured using flow cytometry and adapted from previously described protocols (57, 58). Briefly, MRGPRX2-transfected (or null) HEK293 cells (0.25 × 10^6^/ml) were loaded with Fluo-3 dye (4 μg/ml; Invitrogen) containing 0.02% Pluronic F-127 (Invitrogen) and incubated for 30 min at 37°C in the dark. The cells were then washed and resuspended in Hanks Balanced Salt Solution (HBSS; ThermoFischer) containing 0.01 M HEPES (GE Healthcare) and 175 mM Probenecid (Sigma Aldrich). The MFI was recorded for 30 s to obtain a baseline, followed by treatment with stimulus (gap in the analysis) of either C48/80 or LL-37, with and without ssON. The MFI was then recorded for 2.5 min following stimulus to observe Ca^2+^ influx. Flow cytometry was performed using a BD Fortessa (BD). Analysis was performed using the kinetics tool in the software FlowJo™.

### Mice

BALB/c mice were purchased from Taconic Biosciences A/S, Denmark or from Scanbur, Denmark.

### Behavioral Studies

All behavior analyses were performed sin a controlled environment at 20 to 24°C, 45 to 65% humidity, and during the light 12 h day/night cycle and as previously described (43). Briefly, BALB/c mice (>7 weeks, female) were given a 50 μL intradermal injection into the nape of C48/80 (10 μg), a pre-mixed combination of C48/80 (10 μg) and ssON (2.5 or 25 μg), SLIGRL (100 μg), or a pre-mixed combination of SLIGRL (100 μg) and ssON (2.5 or 25 μg). The mouse behavior was recorded for 60 min and the itch behavior was documented and scored manually, as previously described (43), by observers blind to the treatment and using the software AniTracker v1. A scratching episode was defined as a targeted hind paw scratching toward the pruritogen-injected area from the time-point when the hind paw was lifted until it was placed back on the ground.

### Toluidine Blue Staining

Murine skin biopsies were taken at the injection site ~1 h post treatment with a 50 μL intradermal injection of either C48/80 (10 μg), a pre-mixed combination of C48/80 (10 μg) and ssON (25 μg), or saline into the nape of the neck, and embedded in OCT (ThermoScientific). Biopsies were cut into 8 μM sections using a Cryostat (Leica), transferred onto SuperFrost slides (ThermoFischer), and fixed with ice-cold 100% acetone. Sections were stained as previously described (59, 60). Briefly, sections were then hydrated for 1–5 min in deionized water and stained with freshly prepared Toluidine blue solution (Sigma Aldrich) for 75 s. Sections were rinsed in three consecutive changes of distilled water, dehydrated using 95 and 100% ethanol, submerged in xylene, and mounted using DPX mounting medium (Sigma Aldrich).

### Scoring of Histological Samples

Histological analysis was carried out by two independent observers in a blinded fashion. To grade the level of MC degranulation in each sample, skin sections were assessed and scored at 20X and 40X magnifications. Qualitative evaluations for each sample were then converted into a numerical score using a scale from 0 to 4: a score of 0 was given for an observation of intact mast cells or a clearly intact sample; 1, for mostly intact; 2, for mild degranulation; 3, moderate degranulation; 4, extensive degranulation or a clearly degranulated sample. Hence, each treatment group received two scores per sample based on the observed MC degranulation by each observer, which were then averaged to obtain a single score. Representative pictures of the histological staining for each treatment group were taken using an inverted microscope (Leica DM IRM) at 40X and 63X magnifications and corrected using Photoshop CC (Adobe) for improved contrast and quality.

### Induction of Rosacea-Like Inflammation

Induction of LL-37-mediated rosacea-like inflammation was induced as previously described (4). BALB/c mice (8–11 weeks, female) were treated subcutaneously with 40 μl of either LL-37 (320 μM), a pre-mixed combination of LL-37 (320 μM) and ssON (57 μg), or PBS, twice a day for 2 days for a total of 4 treatments. Photographs were taken after 72 h of observation, which was defined as the clinical endpoint. Skin biopsies (8 mm) were collected either at the clinical endpoint or 1 h after the first injection.

### RNA Extraction and RT-qPCR

Skin biopsies were immediately placed in RNALater (Qiagen) after collection and stored according to the manufacturer's recommendations. RNA was extracted from the skin biopsies using the RNAeasy plus mini kit (Qiagen) according to the manufacturer's recommendations. During the extraction process, the tissue was homogenized using an mTube (Miltenyi Biotech) with the gentleMACS dissociator (Miltenyi Biotech). cDNA was generated using SuperScript™ III Reverse Transcriptase (Invitrogen) according to manufacturer's recommendations. The synthesized cDNA was normalized using a Nanodrop spectrophotometer (Thermo Scientific). Real-time PCR reactions were prepared using the LightCycler® Fast Start DNA Master SYBR® Green (Roche) according to the manufacturer's instructions. The samples were run on a Light Cycler 480 II (Roche) and under the following conditions: initial denaturation step for 10 min at 95°C followed by 45 cycles constituting 10 s at 95°C, 20 s at 50°C, and 20 s at 72°C. Relative gene expression was measured using primers for mouse *Gapdh, Mmp9*, and *Tpsab1* from SinoBiological and primers for *Cma1* from Biorad, according to manufacturer's recommendations. All PCR reactions yielded only a single product species as revealed by melting point analysis. Gene expression was calculated using the delta-delta CT method and as the relative expression to *Gapdh*. All data are presented as fold change of the respective negative control.

### Statistics

All statistical analysis was performed using the GraphPad Prism software. Gaussian distribution was tested using Shapiro-Wilks normality test. One-way ANOVA, with Tukey multiple comparison posttest or Kruskal-Wallis test was used to compare three different treatments. Two independent treatments were compared using either Mann-Whitney test or unpaired, two-tailed student *t*-test for the data sets that passed the normality test. Two-way ANOVA with Bonferroni posttest was used to assess the difference on MC degranulation with and without treatment. All data is presented as the mean ± standard error of the mean (SEM). The number of individuals and repeated experiments is stated in each figure legend.

### Study Approval

The present studies in mice were reviewed and approved by the local ethical committee in Uppsala and the Stockholm North Ethical Committee on Animal Experiments, permit number 12480-2018, and followed the Directive 2010/63/European Union of the European Parliament and of the Council, The Swedish Animal Welfare Act [SFS (Svensk författningssamlingar) 1988:534], The Swedish Animal Welfare Ordinance (SFS 1988:539), and the regulations regarding the use of animals for scientific purposes: SJVFS (Statens jordbruksverks författningssamlingar) 2017:40 (L150).

## Data Availability Statement

The raw data supporting the conclusions of this article will be made available by the authors, without undue reservation.

## Ethics Statement

The present studies in mice were reviewed and approved by the local ethical committee in Uppsala and the Stockholm North Ethical Committee on Animal Experiments, permit number 12480-2018, and followed the Directive 2010/63/European Union of the European Parliament and of the Council, The Swedish Animal Welfare Act [SFS (Svensk författningssamlingar) 1988:534], The Swedish Animal Welfare Ordinance (SFS 1988:539), and the regulations regarding the use of animals for scientific purposes: SJVFS (Statens jordbruksverks författningssamlingar) 2017:40 (L150).

## Author Contributions

AD and A-LS wrote the manuscript. A-LS, ML, GN, AD, SP, HM, and ER designed the research experiments. ER performed the MC experiments. AD, HM, EM, and TG performed the behavioral studies. AD and SP performed the calcium influx experiments. AD, A-LS, and LA were involved in the histological preparation and staining. AD performed all the experiments involving the rosacea mouse model. EL provided MRGPRX2-transfected HEK293 cells. All authors contributed to the writing and review of the manuscript.

## Conflict of Interest

AD and A-LS are shareholders of TIRmed Pharma in possession of intellectual properties related to ssON. The remaining authors declare that the research was conducted in the absence of any commercial or financial relationships that could be construed as a potential conflict of interest.
