# Supplementary material for: Amelioration of Compound 48/80-Mediated Itch and LL-37-Induced Inflammation by a Single-Stranded Oligonucleotide
Source: Front Immunol. 2020 Sep 30;11:559589. doi: 10.3389/fimmu.2020.559589 (PMC7554336; doi:10.3389/fimmu.2020.559589)
Supplement: Supplementary file 1 [file Data_Sheet_1.PDF]

SUPPLEMENTARY FIGURES

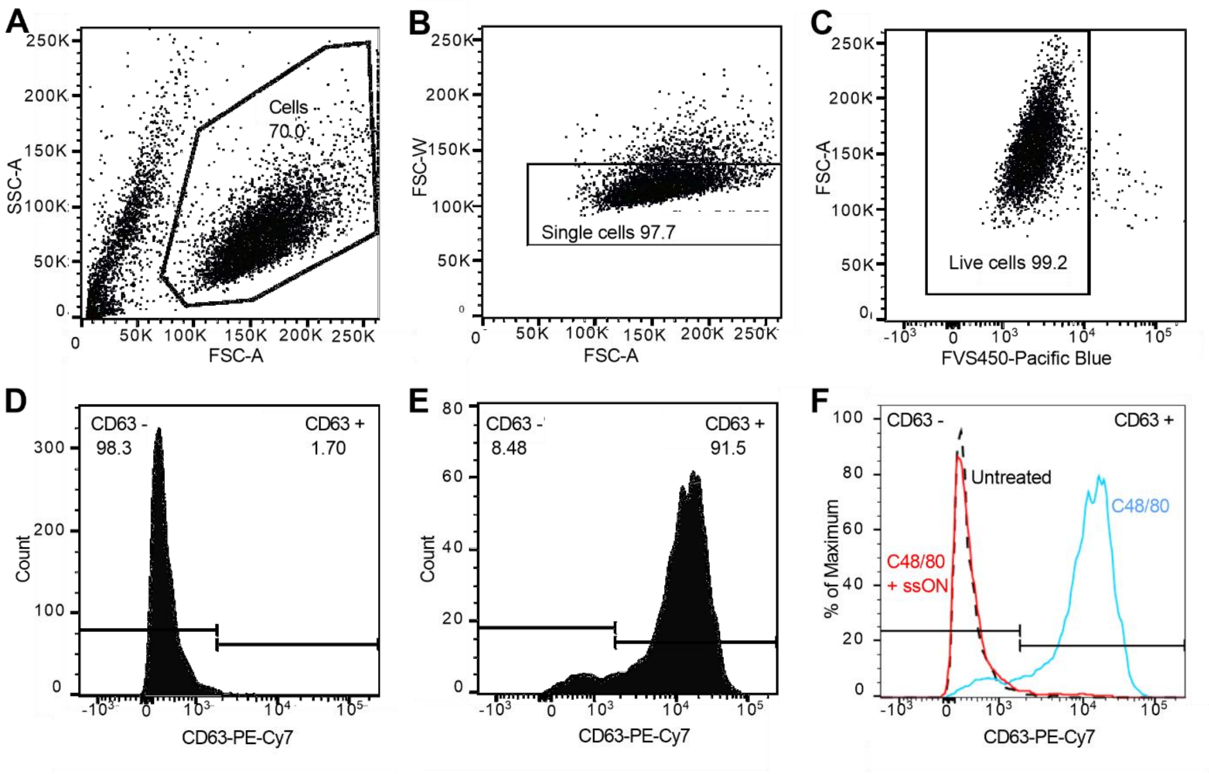

Supplementary Figure 1. Flow cytometry gating strategy for the degranulation of LAD2 cells. (**A**) LAD2 cells were separated from debris based on forward-scattered light (FSC) area and side-scattered light (SSC) area. (**B**) Single cells were then selected using FSC-area versus FSC-width and subsequently, (**C**) dead cells were excluded using Fixable Viability Stain 450 (FVS450). The frequency of degranulated (CD63+) cells was determined using a histogram for CD63-PE-Cy7 expression. Representative gating is shown for (**D**) untreated and (**E**) C48/80 treated cells. (**F**) Overlapping histograms of a representative experiment is also shown; untreated cells are represented as a black dashed line, C48/80 (1 µM) treated cells as a blue line, and the combination treatment of C48/80 (1 µM) and ssON (0.5 µM) is shown as a red line.

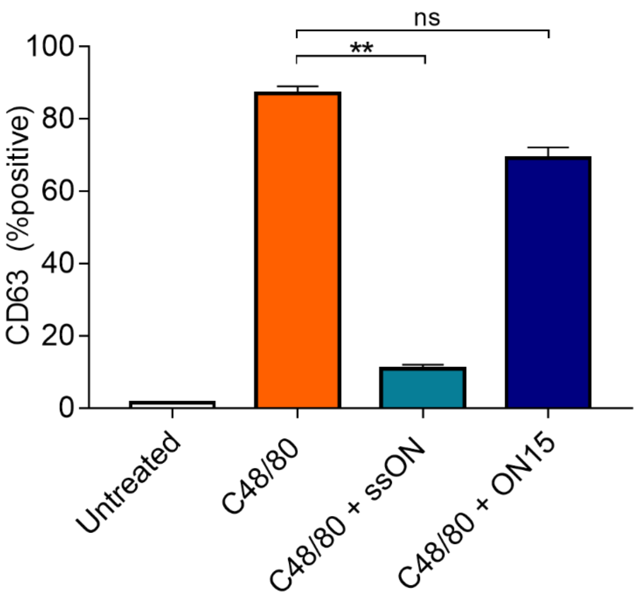

Supplementary Figure 2. C48/80-mediated degranulation was blocked by ssON, but not by the inactive oligonucleotide ON15. Degranulation of LAD2 cells was measured by the upregulation of CD63 on the cell surface. LAD2 cells were stimulated with C48/80 (1.5 µM), with and without ssON or ON15 (0.5 µM) for 30 minutes. Statistics: nonparametric Kruskal-Wallis test. P-value: **P 0.001, ns = non-significant. Data is shown as mean ± SEM. Experiments were performed three times in duplicate.

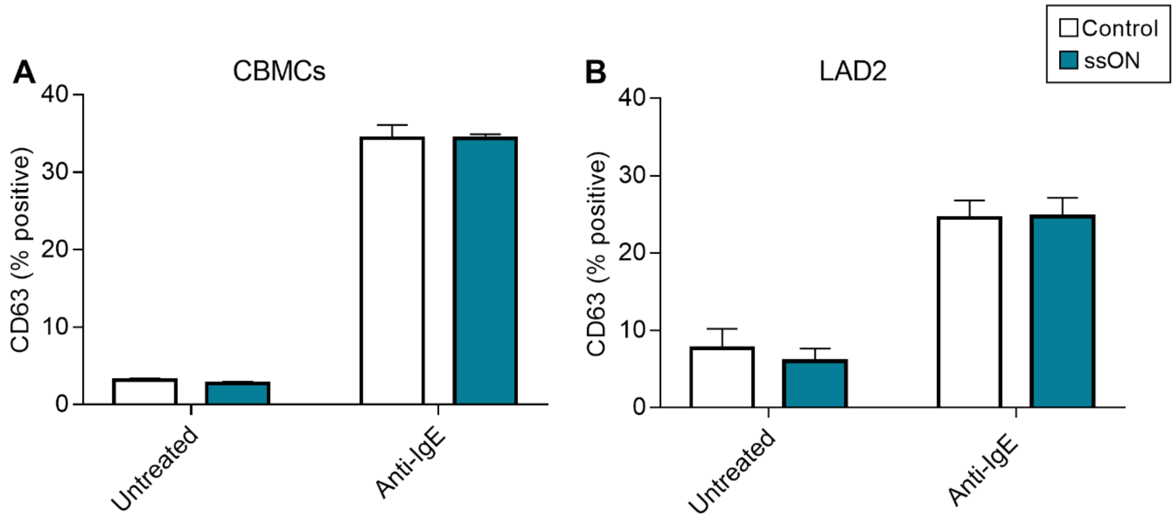

Supplementary Figure 3. ssON had no effect on IgE-mediated MC degranulation. (**A**) Human cord blood-derived MCs (CBMCs) and (**B**) LAD2 cells were stimulated with anti-IgE antibody, with and without ssON (0.5 µM). Experiments were performed at least twice in duplicate. A representative result in shown. Data is shown as mean ± SEM.

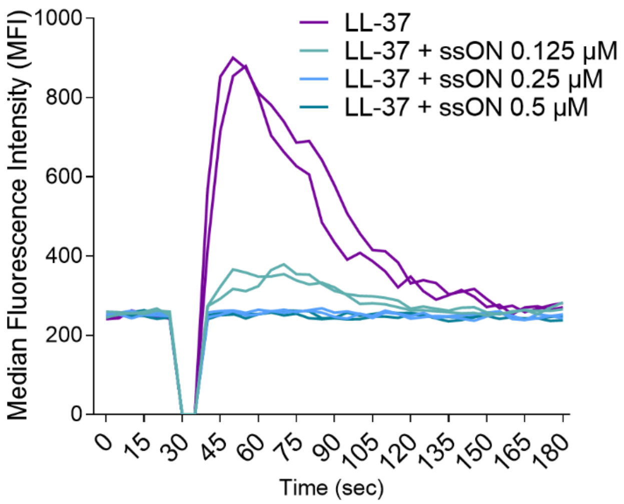

Supplementary Figure 4. Ca^{2+} influx induced by LL-37 is blocked by ssON in a concentration dependent manner. Intracellular calcium (Ca^{2+}) influx in MRGPRX2-transfected HEK293 cells treated with LL-37 (10 µg/ml) is inhibited by ssON in a dose-dependent manner. Ca^{2+} influx was determined by loading cells with Fluo-3 dye and measuring the median fluorescence intensity (MFI) during treatment. MFI curves were recorded for 30 seconds to obtain a baseline. The cells were then stimulated during the gap in the analysis with LL-37(10 µg/ml) and ssON at the various concentrations shown. Each curve represents an independent sample. Gaussian smoothing is applied. The experiment was performed once in duplicate.

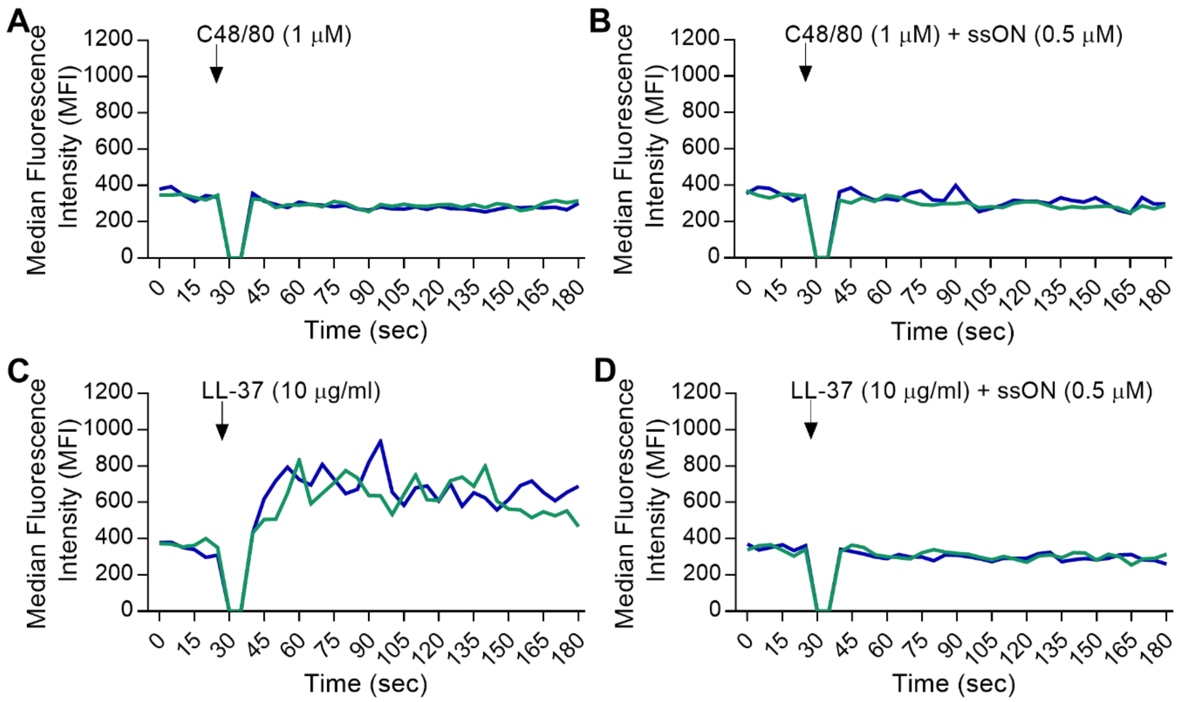

Supplementary Figure 5. C48/80, unlike LL-37, does not induce Ca^{2+} influx in HEK293 cells. A HEK293 cell line was used as a negative control as it does not express MRGPRX2. Intracellular calcium (Ca^{2+}) influx was determined by loading cells with Fluo-3 dye and measuring the median fluorescence intensity (MFI) during treatment. MFI curves were recorded for 30 seconds (sec) to obtain a baseline. The cells were then stimulated during the gap in the analysis with (**A**-**B**) C48/80 or (**C**-**D**) LL-37, with and without ssON, and further recorded for 2.5 minutes. Each curve represents an independent sample. Gaussian smoothing is applied. Two independent experiments were performed in duplicate. A representative result is shown.

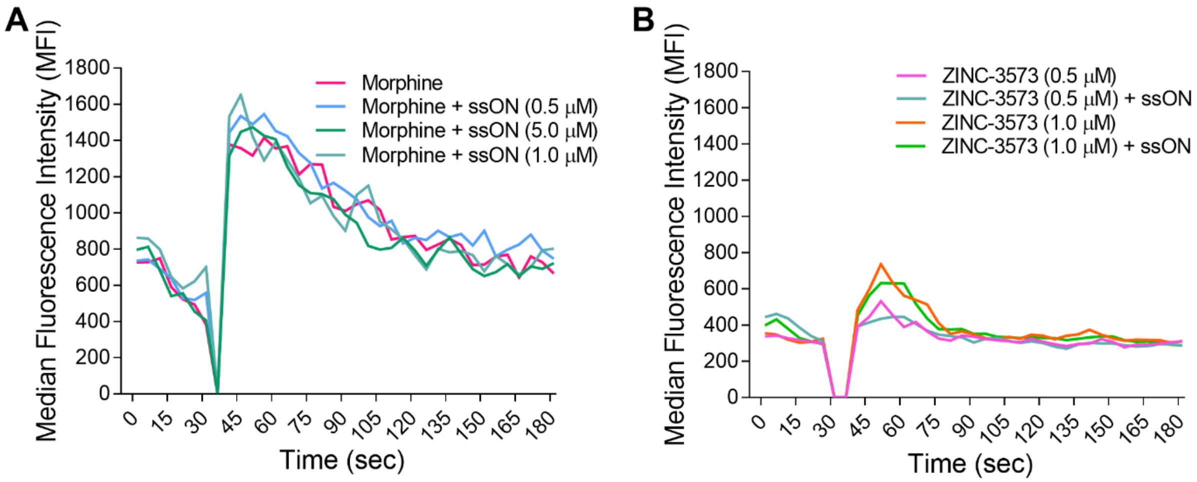

Supplementary Figure 6. Ca^{2+} influx induced by morphine and ZINC-3573 was not affected by ssON treatment. Intracellular calcium (Ca^{2+}) influx in MRGPRX2-transfected HEK293 cells treated with (**A**) morphine (25 μM) with or without ssON at the various concentrations shown, and (**B**) ZINC-3573 with and without ssON (0.5 μM). Ca^{2+} influx was determined by loading cells with Fluo-3 dye and measuring the median fluorescence intensity (MFI) during treatment. MFI curves were recorded for 30 seconds to obtain a baseline. The cells were then stimulated during the gap in the analysis. Each curve represents an independent sample. Gaussian smoothing is applied.
